# Neurobiology of Behavior—Influences of Neuropsychiatric Disorders on Neurobiology and Behavior

**DOI:** 10.3390/biology12060807

**Published:** 2023-06-02

**Authors:** Marc Fakhoury, Michael Fritz

**Affiliations:** 1 Department of Natural Sciences, School of Arts and Sciences, Lebanese American University, Beirut P.O. Box 13-5053, Lebanon; 2 School of Health and Social Sciences, AKAD University of Applied Sciences, 70191 Stuttgart, Germany; 3 Department of Forensic Psychiatry and Psychotherapy, Ulm University, BKH Günzburg, Lindenallee 2, 89312 Günzburg, Germany

The field of neuroscience continues to unravel the mysteries of the human brain and its association with neuropsychiatric disorders. Virtually all psychiatric disorders manifest themselves through deviant patterns that can be observed at a behavioral level. A better understanding of the complex nature of neuropsychiatric disorders and its influence on human behavior is a crucial endeavor in both research and clinical settings. Over the last several decades, advances have been made in isolating factors on a cellular, receptor, neuroanatomical and connectivity level that contribute to the manifestation of clinically recognized pathological behaviors. However, further improvements in knowledge through basic research are essential to understand more aspects of the neurobiological mechanisms underlying the behavioral dimension of psychiatric disorders and to improve their prediction and treatment options. In this Special Issue, we propose transdisciplinary questions of how and why do neuropsychiatric disorders manifest themselves on a behavioral level; what are the archetypical molecular mechanisms underlying them; and how can we create safer and more effective treatments through a common interdisciplinary effort? Studies published in this issue shed light on different aspects of brain function and behavior, offer valuable insights into the underlying mechanisms of neuropsychiatric disorders and discuss various aspects of neuropharmacological effects. The findings of these studies are discussed below.

In the study conducted by Dubuson and colleagues [1], the behavioral response of individuals with alcohol use disorder (AUD) or gambling disorder (GD) to addiction-related cues is investigated using a contextual Go/No-Go task. Findings of the study indicate that AUD patients exhibit poor inhibitory performance, manifested as slower response latencies, lower N2d amplitude and delayed P3d components. In addition, AUD patients show preserved inhibitory performance in the alcohol-related context, whereas GD patients display a specific inhibitory deficit in the gambling-related context. These findings suggest that despite sharing common addiction-related mechanisms, AUD and GD patients have different responses to rewarding cues. The implementation of personalized treatment approaches tailored to each disorder and taking into account specific context and cues is therefore of primordial importance.

In another study, Tanaka and colleagues [2] explore the effects of a diet including soybean sprouts rich in the HASPIN inhibitor coumestrol on a mouse model of Alzheimer’s disease (AD). HASPIN is a serine/threonine kinase expressed in various cells that is thought to be involved in tau protein phosphorylation. The findings of the study show that ingestion of soybean sprouts containing coumestrol suppresses the development of spatial cognitive dysfunction in AD mice, indicating that HASPIN may serve as a potential therapeutic target for the treatment of AD. These findings open new avenues for investigating the clinical application of HASPIN inhibitors in humans, warranting further research and investigation in the hunt for more effective treatment strategies for AD.

In the study conducted by Ahn and colleagues [3], the function of top-down sensory prediction error and efference copy (EC) signals to investigate the effects of peripheral hearing loss on auditory false perception and phantom perception is examined. Patients in the study have either schizophrenia or tinnitus accompanied by varied degrees of hearing loss. Findings show that auditory responses to self-generated sound were not reduced in either group of patients. However, attenuated auditory responses were similar to those of healthy controls in patients with tinnitus and mild hearing impairment. These results imply that EC signals from auditory deafferentation may affect the sensorimotor integration process. Importantly, the study shows that individuals with tinnitus and substantial hearing loss maintain their auditory attention networks to self-generated sound, suggesting that the impaired sensorimotor integration may have resulted from deficits in auditory signals and not in the reafference system. Findings of this study could provide valuable insights for future investigations, opening the door for potential therapeutic interventions.

In the study conducted by Ulrich and colleagues [4], researchers investigate how attention deficit hyperactivity disorder (ADHD) patients’ intrinsic functional connectivity (IFC) changed after receiving therapy with methylphenidate (MPH). The study looked at brain areas with changes in IFC linked to a decrease in ADHD symptoms using resting-state functional magnetic resonance imaging (rs-fMRI) and a seed-based approach. Findings show that only MPH treatment responders are able to observe drug effects on IFC and demonstrate significant changes in the IFC of bilateral putamen in response to medication. The novel analytical framework and results presented in this study show the potential of seed-based rs-fMRI analysis in clarifying changes in IFC related to symptom reduction in addition to providing supported evidence for the role of the striatum in the treatment of adult ADHD with MPH. A more thorough comprehension of the neurobiological mechanisms underlying the pharmaceutical effects of MPH in adult ADHD may ultimately aid in the creation of individualized treatment plans and improved patient outcomes.

Also published in this Special Issue is a study conducted by Dousset and colleagues [5] that investigates the cognitive function of reactive inhibition in (poly)drug users undergoing a detoxification program. By employing a “contextual Go/No-Go task” combined with electroencephalography, the researchers compare the performance of cocaine users, heroin users, polydrug users and healthy controls. Results reveal that polydrug users exhibit a higher rate of commission errors compared with the control group, indicating impaired inhibitory function. Additionally, the study shows that impaired performance monitoring and error-processing could contribute to impaired awareness in cocaine users, thereby preventing these individuals from changing their behaviors. These findings highlight the detrimental impact of drug use on inhibitory functions and provide insights into the cognitive mechanisms underlying addictive disorders. Findings also emphasize the urgent need for targeted interventions and prevention strategies to address the global increase in polydrug use, which could ultimately pave the way for improved treatment initiatives in addictive disorders.

In another study, Chithanathan and colleagues [6] investigate symptoms of anxiety and neuroinflammation in a mouse model of familial AD (5 × FAD mice). The study found that AD mice have higher mRNA levels of pro-inflammatory cytokines at an early age in the olfactory bulb, which is accompanied by increased anxiety. In particular, anxiety behavior in the AD mice is substantially linked with tumor necrosis factor (Tnf) expression in the olfactory bulb. In addition, the olfactory bulb of the AD mice exhibit structural abnormalities and increased microglial activation, suggesting the involvement of neuroinflammation in anxiety-related processes. Altogether, the findings of this study support the notion that the olfactory bulb could serve as an early stage biomarker for AD. These findings could open avenues for additional investigation and examination of neuroinflammatory pathways and therapeutic strategies that aim to alleviate anxiety within the framework of neurodegenerative illnesses.

This Special Issue also includes a study conducted by Zernig and colleagues [7] which investigates the effects of cohabitation on stress levels and social interactions in rodents. Fecal corticosterone and metabolite levels are used as measures of stress levels in two different strains of mice (C57BL/6J and CD1) and in Sprague Dawley rats. Findings of the study show that co-housing C57BL/6J mice with rats increases stress levels compared with solitary-housed or postpartum mice. The results suggest that the effects of rat–mouse cohabitation on stress levels and behavior may be subtler than previously thought. A better understanding of these dynamics could optimize resources for better housing research animals in animal facilities.

In another original research article, Zahr and colleagues [8] focus on the effects of systemic administration of the TLR7/8 agonist Resiquimod (R848) in mice at the neurochemical and morphological levels. Researchers of the study observed transient brain swelling after immune activation and were able to demonstrate a link between peripheral immune stimulation and changes in brain volume. The study also sheds light on the evolution of peripheral immune stimulation effects over time, suggesting that neurochemical changes occur faster than neurostructural changes. A better understanding of the complex interplay between peripheral immune activation and central nervous system alterations is crucial, as it could help elucidate the underlying mechanisms of inflammatory and neurological diseases.

The study by Gouda and colleagues [9] uses a mouse model to investigate the role of gender differences in the development of autism spectrum disorders (ASD). Specifically, this study examines the morphological and behavioral changes induced by valproic acid (VPA) administration in postnatal BALB/c mice. Findings of the study reveal significant differences in brain histology of male and female mice and suggest that male animals are more susceptible to VPA-induced ASD than females. Observed differences at the neurochemical and behavioral levels highlight the importance of understanding the sex-specific aspects of ASD. A better understanding of the mechanisms underlying such gender differences in ASD may contribute to the development of targeted interventions and treatments for ASD, ultimately aiming to improve the lives of affected individuals.

The article by Tisserand and colleagues [10] focuses on the role of the insular cortex in self-awareness and provides a comprehensive overview of the anatomical and functional aspects of this brain region. The insula is a highly interconnected region that plays a key role in integrating body-state and self-related information. There have been several examples of evidence supporting the idea that damage to the insular cortex can have profound effects on the self, including impaired autobiographical memory. However, the exact mechanisms underlying the relationship between the insula to the self require further investigation. Future work in this field could hold the potential to further strengthen our understanding of the anatomical substrates of the self and could contribute to a more comprehensive framework for addressing the global collapse of self-experience characterizing different neurological and psychiatric disorders.

The urgent problem of aggressive behavior and its connection to alcohol and trauma is covered in detail in the review paper by Fritz and colleagues [11]. The many facets of aggression are influenced by a number of variables, such as heredity, mental health issues, socioeconomic situation and substance misuse. This paper emphasizes the connection between alcohol use and aggressive behavior, and discusses how alcohol contributes to the progression of aggressive behavior and violent crimes. The effects of trauma and posttraumatic stress disorder (PTSD) on aggressive behavior and alcohol consumption are also covered. Although there is ample evidence of a connection between trauma, alcohol and aggression, the underlying neurological pathways are still not fully understood. By learning more about the translational neurobiology of aggression, alcoholism and trauma, future studies may open the door for the development of specialized interventions and therapeutic approaches that address these urgent public health issues.

The chronic unpredictable mild stress (CUMS) model of depression, one of the often employed animal models in depression research, is the subject of the article published by Markov and Novosadova [12]. In their paper, the authors discuss the issue of this model’s low reproducibility and look into potential causes of this variability. They point to diverse combinations of stressors, prolonged sleep deprivation, painful stressors, social stress, changes in animal sex and age, strain-specific stress susceptibility and handling quality as plausible causes of the poor repeatability. The authors also urge researchers to carefully analyze and regulate these variables by emphasizing these parameters in order to improve the reproducibility and validity of studies using the CUMS model. Such advancements will ultimately make it easier to interpret and compare results between different studies

Last but not least, the review paper by Rabat and colleagues [13] examines the possibility of MRI as a technique for locating neuro-biomarkers that can forecast smoking cessation treatment outcomes. The review provides a summary of the findings of MRI-based studies and emphasizes methodological points for future research. Notably, the insula, a part of the brain associated with addiction, is highly discussed. The identification of biomarkers that predict treatment outcomes could eventually improve tailored therapy and help combat addiction, which is a major global health concern worldwide.

Altogether, the articles covered in this Special Issue provide crucial and novel insights about the neurobiological bases of several neurological disorders and conditions including ADHD, ASD, AD, depression and addiction. They also emphasize the significance of taking into account the intricate interactions of genetic, environmental and neurobiological factors that influence behavior and the impact of existing neuropsychiatric disorders. Furthermore, these studies highlight the need for additional investigations to better understand the complex mechanisms involved and to translate recent findings into effective treatment approaches in humans. We believe that the research presented in this Special Issue represents a significant step forward in our quest to comprehend and address the complexities of neuropsychiatric disorders. By strengthening our knowledge of the underlying neurobiology of behavior and the influence of neuropsychiatric disorders, studies included in this Special Issue may open avenues for individualized strategies that cater to the particular requirements of individuals struggling with mental health issues.

## References

[1] Dubuson M., Noël X., Kornreich C., Hanak C., Saeremans M., Campanella S. (2023). A Comparative Event-Related Potentials Study between Alcohol Use Disorder, Gambling Disorder and Healthy Control Subjects through a Contextual Go/NoGo Task. Biology.

[2] Tanaka H., Matsushita H., Tokuhiro K., Fukunari A., Ando Y. (2023). Ingestion of Soybean Sprouts Containing a HASPIN Inhibitor Improves Condition in a Mouse Model of Alzheimer’s Disease. Biology.

[3] Ahn M.-H., Alsabbagh N., Lee H.-J., Kim H.-J., Jung M.-H., Hong S.-K. (2022). Neurobiological Signatures of Auditory False Perception and Phantom Perception as a Consequence of Sensory Prediction Errors. Biology.

[4] Ulrich M., Heckel K., Kölle M., Grön G. (2022). Methylphenidate Differentially Affects Intrinsic Functional Connectivity of the Salience Network in Adult ADHD Treatment Responders and Non-Responders. Biology.

[5] Dousset C., Chenut C., Kajosch H., Kornreich C., Campanella S. (2022). Comparison of Neural Correlates of Reactive Inhibition in Cocaine, Heroin, and Polydrug Users through a Contextual Go/No-Go Task Using Event-Related Potentials. Biology.

[6] Chithanathan K., Xuan F.-L., Hickey M.A., Tian L. (2022). Enhanced Anxiety and Olfactory Microglial Activation in Early-Stage Familial Alzheimer’s Disease Mouse Model. Biology.

[7] Zernig G., Ghareh H., Berchtold H. (2022). Effects of Cohousing Mice and Rats on Stress Levels, and the Attractiveness of Dyadic Social Interaction in C57BL/6J and CD1 Mice as Well as Sprague Dawley Rats. Biology.

[8] Zahr N.M., Zhao Q., Goodcase R., Pfefferbaum A. (2022). Systemic Administration of the TLR7/8 Agonist Resiquimod (R848) to Mice Is Associated with Transient, In Vivo-Detectable Brain Swelling. Biology.

[9] Gouda B., Sinha S.N., Chalamaiah M., Vakdevi V., Shashikala P., Veeresh B., Surekha V.M., Kasturi V., Boiroju N.K. (2022). Sex Differences in Animal Models of Sodium-Valproate-Induced Autism in Postnatal BALB/c Mice: Whole-Brain Histoarchitecture and 5-HT2A Receptor Biomarker Evidence. Biology.

[10] Tisserand A., Philippi N., Botzung A., Blanc F. (2023). Me, Myself and My Insula: An Oasis in the Forefront of Self-Consciousness. Biology.

[11] Fritz M., Soravia S.-M., Dudeck M., Malli L., Fakhoury M. (2023). Neurobiology of Aggression—Review of Recent Findings and Relationship with Alcohol and Trauma. Biology.

[12] Markov D.D., Novosadova E.V. (2022). Chronic Unpredictable Mild Stress Model of Depression: Possible Sources of Poor Reproducibility and Latent Variables. Biology.

[13] Rabat Y., Chanraud S., Abdallah M., Sibon I., Berthoz S. (2022). Precision Preventive Medicine of Relapse in Smoking Cessation: Can MRI Inform the Search of Intermediate Phenotypes?. Biology.

